# Assessing the association between the non-HDL to HDL cholesterol ratio and NAFLD in Chinese adults: concentrate on gout populations

**DOI:** 10.3389/fnut.2025.1655817

**Published:** 2025-09-01

**Authors:** Cunwei Sun, Ke Si, Youzhuang Zhu, Chengqian Li, Yang Yu, Changxin Jia, Qing Wang

**Affiliations:** ^1^Department of Endocrinology and Metabolism, Affiliated Hospital of Qingdao University, Qingdao, China; ^2^Department of Anesthesiology, Affiliated Hospital of Qingdao University, Qingdao, China; ^3^Department of Laboratory Medicine, Affiliated Hospital of Qingdao University, Qingdao, China

**Keywords:** gout, NAFLD, NHHR, lipid indicator, non-HDL, HDL, cross-sectional study

## Abstract

**Background:**

The coexistence and synergistic relationship between Nonalcoholic fatty liver disease (NAFLD) and gout necessitate an investigation into the risk factors for NAFLD among individuals with gout. The non-high-density lipoprotein cholesterol to high-density lipoprotein cholesterol ratio (NHHR) serves as a comprehensive lipid index. This study aimed to investigate the association between NHHR and the risk of NAFLD in patients with gout.

**Methods:**

A cross-sectional study was conducted involving 1,038 hospitalized patients with gout to examine the relationship between NHHR and NAFLD. NHHR was included in the logistic regression analysis as both a continuous and a categorical variable. Restricted cubic splines (RCS) were utilized to assess the dose–response relationship. Additionally, subgroup analyses were performed to identify potential interactions among variables. The predictive capability of NHHR was evaluated using the receiver operating characteristic (ROC) curve based on the basic model.

**Results:**

The analysis of quartile groups stratified by NHHR levels revealed an increased prevalence of NAFLD corresponding to higher NHHR levels. Multifactorial logistic regression analysis established a significant association between NHHR and NAFLD, yielding an odds ratio (OR) of 1.242 [95% confidence interval (CI): 1.089–1.416, *p* = 0.001]. When treated as a categorical variable, the OR for NHHR in the fourth quartile was significantly elevated compared to the lowest quartile, with values of 1.993 (95% CI: 1.349–2.944, *p* = 0.001). The RCS analysis demonstrated a non-linear dose–response relationship between NHHR and NAFLD across all models. No significant interactions were detected in the subgroup analysis. Incorporating NHHR into the basic model enhanced the area under the curve (AUC) of the ROC curve to 0.706.

**Conclusion:**

This study identified a positive correlation between NHHR and the incidence of NAFLD in individuals with gout, suggesting that NHHR may serve as a reliable indicator of NAFLD within the gout patient.

## 1 Introduction

Gout is often the result of monosodium urate crystals accumulating in tissues both within and outside of joints. Elevated urate levels in the bloodstream are the main risk factor for gout (1). The prevalence of gout among adults has been reported to range from 0.68 to 3.90% in studies from Asia, Europe, and North America (1). The prevalence of gout in China is also between 0.4 and 1.5%, according to a systematic review (2). With its increasing frequency among the global population, gout is evolving into a significant issue for public health.

Nonalcoholic fatty liver disease (NAFLD), which includes conditions from simple liver fat buildup to nonalcoholic steatohepatitis, is the primary cause of chronic liver disease globally, affecting over 20% of the global population (3, 4). NAFLD has a significant connection with gout. NAFLD considerably elevates the chance of hyperuricemia, which is the main contributor to gout (5). Our previous research also demonstrates that frequent gout flares have a strong predictive ability for the development of NAFLD (6). Gout is a prevalent and intensely painful form of inflammatory arthritis. The long-term management of patients with gouty arthritis primarily involves uric acid-lowering therapy to mitigate hyperuricemia and consequently prevent gout attacks (7, 8). However, there has been limited attention to the complications associated with gout. Furthermore, gout and NAFLD frequently co-occur and may exacerbate each other’s effects. The diagnosis of NAFLD remains a process of exclusion, requiring evidence of hepatic fat accumulation through imaging or histological analysis, while simultaneously ruling out other potential causes such as alcohol consumption (9). Therefore, it is crucial to identify the predictors and risk factors independently associated with NAFLD in patients with gout.

Numerous studies indicate that the accumulation of lipids in the liver contributes to the development of NAFLD (10, 11). Specifically, NAFLD involves lipid accumulation in hepatocytes, which leads to steatosis, inflammation, and fibrosis (12). Prior investigations have revealed that numerous lipid indicators, like LDL-C, residual cholesterol, and HDL-C, are closely linked to the development of NAFLD (13, 14). The non-high-density lipoprotein cholesterol to high-density lipoprotein cholesterol ratio (NHHR), which stands for the non-high-density lipoprotein cholesterol to high-density lipoprotein cholesterol ratio, is a newly discovered marker for analyzing lipid composition in atherosclerosis (15). In comparison with traditional lipid parameters, it illustrates the dual effects of non-HDL-C and HDL-C, circumventing the limitations of prior monolipid research. NHHR is related to different diseases and offers predictive value, including conditions like depression, kidney stones, and cardiovascular diseases (16–18). NHHR is more effective in diagnosis and prediction than traditional lipid markers. Additionally, elevated NHHR levels in American adults are connected to a higher probability of gout (15). NHHR may act as a significant metabolic bridge between gout, dyslipidemia, and NAFLD. However, there is not enough evidence to support a link between NHHR and the risk of NAFLD in people with gout.

In this research, we conducted a cross-sectional analysis on individuals with gout. This research sought to analyze the association between NHHR and NAFLD among gout patients, assessing NHHR as a possible predictive biomarker. These discoveries might enable the formulation of preventive and therapeutic solutions.

## 2 Materials and methods

### 2.1 Study design and participant details

**Figure 1:**
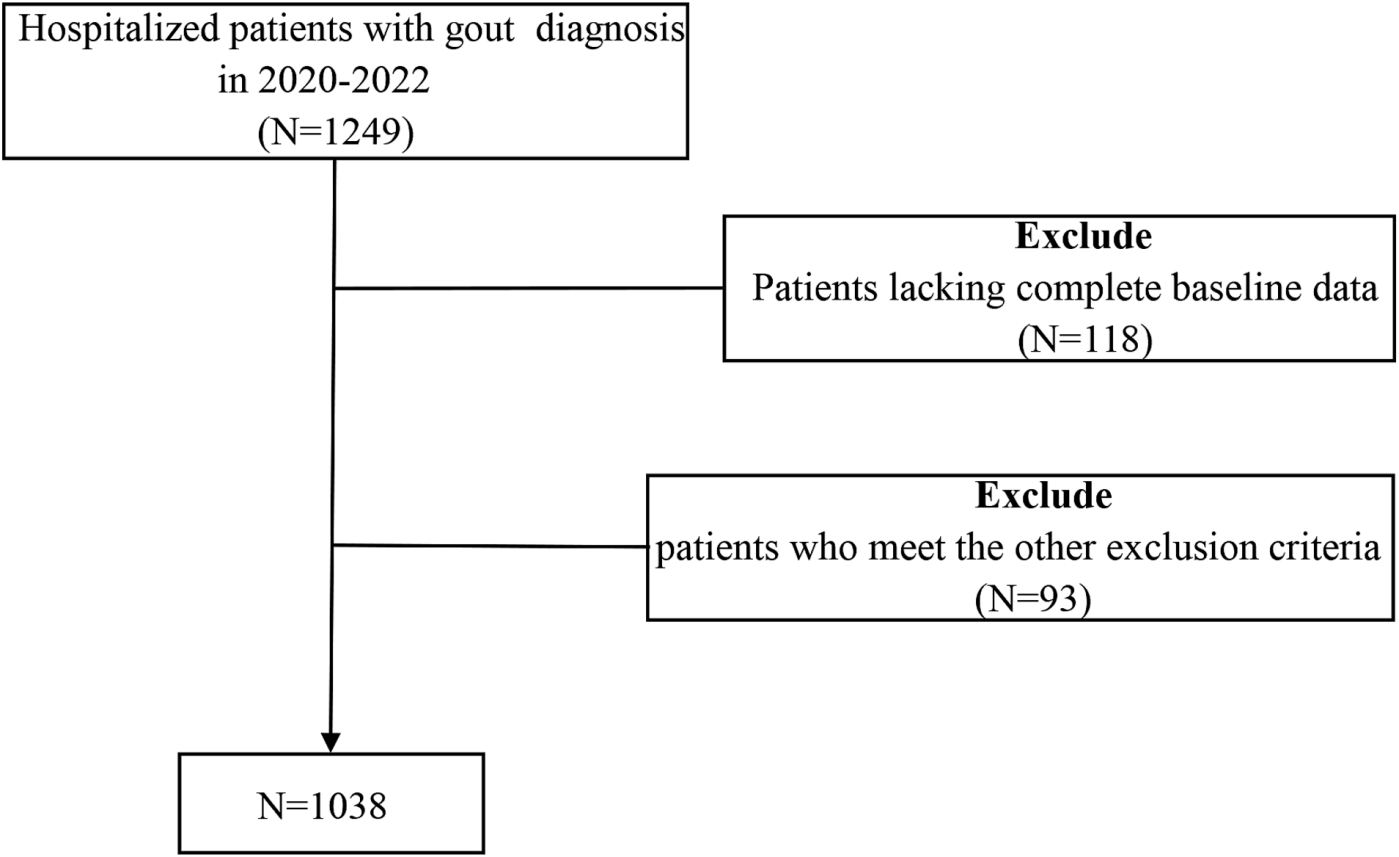
Flowchart of the study population.

The study was observational and used a cross-sectional approach, and data were gathered from 1,038 patients who were hospitalized at the Affiliated Hospital of Qingdao University from 2017 to 2019. The inclusion criteria include: (1) meeting the 2015 ACR/EULAR diagnostic standards for gout; (2) being at least 18 years of age; and (3) possessing comprehensive clinical data. The criteria for exclusion were: (1) individuals under 18 years old; (2) a history of alcohol abuse; (3) those undergoing lipid-lowering treatment; (4) simultaneous acute or chronic infections; (5) the existence of cancerous tumors or autoimmune disorders; and (6) any dysfunction or failure of organs. The selection procedure for study participants is depicted in Figure 1. Approval for this study was granted by the Affiliated Hospital of Medical College Qingdao University (QYFY WZLL 30269). The methods detailed here adhered to relevant guidelines and regulations, and all individuals involved signed consent forms that were written and informed.

### 2.2 Data collection

Collected baseline information included the patient’s age, sex, duration of gout, smoking and alcohol use history, weight, systolic/diastolic blood pressure (SBP/DBP), height, and medications taken upon admission. Standard methods were used to measure height, weight, and blood pressure. The formula for calculating body mass index (BMI) is BMI = weight (kg) / height (m^2^).

Venous blood samples were collected post an 8-h fast to evaluate low-density lipoprotein cholesterol (LDL-C), triglycerides (TG), C-reactive protein (CRP), high-density ipoprotein cholesterol (HDL-C), *γ*-glutamyltransferase (GGT), alanine aminotransferase (ALT), total cholesterol (TC), aspartate aminotransferase (AST), alkaline phosphatase (ALP), fasting plasma glucose (FPG), creatinine (Cr), urea nitrogen (BUN), urine microalbumin/creatinine (UACR), uric acid (UA), thyroid-stimulating hormone (TSH), free thyroid hormone (FT4), and free triiodothyronine (FT3). The calculation of glomerular filtration rate (eGFR) was performed with the Chronic Kidney Disease Epidemiology Collaboration (CKD-EPI) equation.

The NHHR served as an independent variable for assessing exposure, determined by the ratio of non-HDL-C to HDL-C levels (NHHR = non-HDL-c/HDL-c). Non-HDL-C is calculated by taking TC and subtracting HDL-C (Non-HDL-C = TC - HDL-C). TC and HDL-C information was derived from lab data. Four separate quartiles were used to divide the NHHR values: Quartile 1 (<2.69), Quartile 2 (2.70–3.33), Quartile 3 (3.34–4.07), and Quartile 4 (>4.08). NAFLD was diagnosed by observing liver fat accumulation without significant alcohol consumption or other chronic liver diseases (9).

### 2.3 Statistical methods

SPSS 27.0 and R software (version 4.3) were utilized for conducting the statistical analysis. The normality of continuous variables was evaluated based on NHHR quartiles. For normally distributed data, the expression used was the mean ± standard deviation, and the ANOVA test was employed for group comparisons. For skewed data, the median and the interquartile range (25th to 75th percentile) were shown, and group comparisons utilized the Kruskal-Wallis H test. Categorical variables were shown as percentages, with Pearson chi-square tests used for comparing groups. Findings were deemed statistically significant if the *p*-value was 0.05 or lower (*p* < 0.05).

To lessen the effect of confounding factors, potential confounders underwent systematic screening. Multifactorial logistic regression was employed to study the association between NHHR and NAFLD. To shed more light on these associations, three models using multivariable logistic regression were constructed. Model 1 did not include any covariate adjustments. Model 2 considered factors like age, and Model 3 added adjustments for UA, eGFR, phosphorus, and magnesium. The median NHHR values within each quartile were added to the model, followed by the computation of the *p*-trend. To further investigate the relationship between NHHR and NAFLD, a restricted cubic spline (RCS) analysis with logistic regression was conducted. After recognizing nonlinearity, a piecewise linear relationship was established using segmented regression. The threshold for the inflection point was identified using a recursive algorithm. To assess the consistency of the findings across various stratifications such as age, BMI, and UA, we conducted a subgroup analysis. Furthermore, we explored the interaction of these stratified variables with NHHR. To determine the prediction accuracy of the baseline model compared to the NHHR-enhanced model for NAFLD, we employed Receiver Operating Characteristic (ROC) curves.

## 3 Results

### 3.1 Baseline characteristics divided by NHHR quantiles

The study included 1,038 participants, with 1,015 (97.8%) being male and 23 (2.22%) females, and their mean age was 50.39 ± 14.87 years. This population included 623 (60.0%) participants with NAFLD and 415 (40.0%) without. Participants were classified into four different groups by the NHHR through quantile analysis (Table 1). The high-NHHR quantile group exhibited significantly elevated levels of BMI, TC, LDL-C, TG, eGFR, UA, ALT, GGT, and phosphorus (*p* < 0.05). Conversely, the group with a high NHHR quantile was significantly younger (*p* < 0.001). Notably, an increased NHHR correlated with a greater occurrence of NAFLD, with prevalence rates of 46.3, 61.2, 64.5, and 68.0% (*p* < 0.001).

**Table 1:** The general characteristics of the gout patients grouped by NHHR quartiles.

Variables	Total (*n* = 1,038)	NHHR				Effect size	*p* value
		Q1 (≤2.69)	Q2 (2.70–3.33)	Q3 (3.34–4.07)	Q4 (≥4.08)		
		*n* = 259	*n* = 258	*n* = 262	*n* = 259		
Age (year)	50.39 ± 14.871	52.91 ± 15.112	51.54 ± 15.169	50.14 ± 14.471	46.98 ± 14.143	0.022^a^	<0.001
Sex: Male (%)	97.8% (1,015)	97.3% (252)	96.5% (249)	98.1% (257)	99.2% (257)	0.068^b^	0.186
Duration of gout(year)	6 (3–10)	8 (3–13)	6 (3–10)	6 (3–10)	6 (2–10)	0.002^a^	0.194
Smoking (%)	50.0% (517)	45.3% (117)	51.4% (132)	53.3% (139)	49.8% (129)	0.058^b^	0.316
BMI(kg/m^2^)	27.70 ± 3.947	26.78 ± 3.877	27.58 ± 3.946	27.65 ± 3.705	28.78 ± 4.018	0.033^a^	<0.001
SBP (mmHg)	136.40 ± 18.134	138.29 ± 21.152	135.49 ± 17.08	134.90 ± 17.812	136.93 ± 15.979	0.007^a^	0.141
DBP (mmHg)	84.80 ± 11.92	85.27 ± 12.425	84.37 ± 12.128	83.75 ± 11.932	85.81 ± 11.121	0.004^a^	0.186
CRP(mg/L)	10.20 (3.44–33.70)	13.40 (3.44–43.20)	11.00 (3.44–28.63)	9.92 (3.44–33.10)	8.06 (3.44–28.50)	0.002^a^	0.145
FPG (mmol/L)	5.05 ± 0.897	5.06 ± 0.871	5.05 ± 0.948	4.96 ± 0.756	5.13 ± 0.993	0.005^a^	0.179
TC(mmol/L)	4.74 (4.11–5.42)	4.12 (3.62–4.53)	4.60 (4.10–5.345)	4.89 (4.44–5.43)	5.38 (4.80–6.08)	0.205^a^	<0.001
LDL-C(mmol/L)	2.91 ± 0.782	2.32 ± 0.575	2.80 ± 0.640	3.06 ± 0.577	3.46 ± 0.835	0.279^a^	<0.001
HDL-C(mmol/L)	1.12 ± 0.271	1.34 ± 0.291	1.17 ± 0.236	1.05 ± 0.177	0.94 ± 0.187	0.299^a^	<0.001
TG (mmol/L)	1.54 (1.09–2.32)	1.09 (0.83–1.49)	1.47 (1.09–2.05)	1.66 (1.28–2.27)	2.20 (1.55–3.34)	0.196^a^	<0.001
eGFR (mL/min/1.73 m^2^)	91.48 ± 29.001	88.93 ± 29.598	89.54 ± 28.038	91.54 ± 29.507	95.91 ± 28.469	0.0092^a^	0.026
UA (umol/L)	483.61 ± 124.960	459.51 ± 114.945	471.72 ± 123.432	489.21 ± 126.166	513.90 ± 128.816	0.027^a^	<0.001
ALT(U/L)	44.60 ± 45.548	36.56 ± 42.018	43.74 ± 47.649	49.05 ± 49.541	48.93 ± 41.512	0.013^a^	0.005
AST(U/L)	24.49 ± 17.763	22.68 ± 17.955	23.95 ± 20.061	25.68 ± 16.697	25.60 ± 16.024	0.005^a^	0.164
GGT(U/L)	52.27 ± 51.958	45.03 ± 49.574	48.38 ± 47.632	55.19 ± 48.407	60.45 ± 60.184	0.013^a^	0.003
ALP (U/L)	76.27 ± 28.440	73.44 ± 29.459	75.12 ± 23.639	76.39 ± 26.764	80.23 ± 32.837	0.008^a^	0.052
TSH(mIU/L)	1.89(1.15–2.96)	1.86(1.10–2.84)	1.82(1.04–2.84)	1.93(1.35–3.09)	1.98(1.25–3.09)	-0.001^a^	0.553
FT4(pmol/L)	16.47 ± 2.781	16.35 ± 2.635	16.66 ± 2.894	16.16 ± 2.582	16.70 ± 2.973	0.007^a^	0.106
FT3(pmol/L)	4.58 ± 0.912	4.47 ± 0.956	4.61 ± 0.954	4.59 ± 0.873	4.66 ± 0.855	0.006^a^	0.118
Phosphorus (mmol/L)	1.21 ± 0.202	1.19 ± 0.208	1.19 ± 0.192	1.21 ± 0.176	1.27 ± 0.220	0.028^a^	<0.001
Magnesium (mmol/L)	0.92 ± 0.104	0.92 ± 0.098	0.91 ± 0.110	0.94 ± 0.098	0.907 ± 0.110	0.011^a^	0.012
Sodium (mmol/L)	141.66 ± 6.732	141.87 ± 2.426	141.61 ± 9.148	142.11 ± 2.849	141.05 ± 9.178	0.008^a^	0.316
Potassium (mmol/L)	4.34 ± 0.411	4.34 ± 0.460	4.33 ± 0.395	4.36 ± 0.378	4.31 ± 0.406	0.002^a^	0.638
Kidney stones	15.5% (161)	14.7% (38)	12.4% (32)	14.9% (39)	20.1% (52)	0.078^b^	0.101
Febuxostat	51.0% (529)	51.7% (134)	49.2% (127)	52.3% (137)	50.6% (131)	0.024^b^	0.902
Allopurinolo	5.8% (60)	6.9% (18)	5.0% (13)	6.9% (18)	4.2% (11)	0.050^b^	0.457
NAFLD	60.0% (623)	46.3% (120)	61.2% (158)	64.5% (169)	68.0% (176)	0.168^b^	<0.001
Benzbromarone	15.1% (157)	15.1% (39)	15.9% (41)	13.4% (35)	16.2% (42)	0.031^b^	0.802

BMI, body mass index; SBP, systolic blood pressure; DBP, diastolic blood pressure; HbA1c, glycated hemoglobin; FPG, fasting plasma glucose; TC, total cholesterol; LDL-C, low-density lipoprotein cholesterol; HDL-C, high-density lipoprotein cholesterol; TG, triglycerides; UACR, urine microalbumin/creatinine; eGFR, estimated glomerular filtration rate; UA, uric acid; ALT, alanine aminotransferase; AST, aspartate aminotransferase; GGT, γ-glutamyltransferase; ALP, alkaline phosphatase; TSH, thyroid-stimulating hormone; FT4, free thyroid hormone; and FT3, free triiodothyronine; ^a^Eta Squared; ^b^Cramer’s V.

### 3.2 Investigate the elements that could elevate the likelihood of NAFLD in individuals with gout

Researchers utilized logistic regression to examine the risk factors linked to NAFLD in gout patients. Table 2 provides a summary of the potential risk factors. Our study found a positive association between BMI, DBP, TC, LDL-C, TG, eGFR, UA, ALT, AST, GGT, FT3, phosphorus, magnesium, and NHHR with NAFLD, indicated by odds ratios (OR) over 1 and *p*-values under 0.05. Factors like drinking, febuxostat, age, duration of gout, CRP, and ALP were negatively related to NAFLD, with OR values under 1 and *p*-values less than 0.05. Many lipid-related indicators were associated with NAFLD in patients with gout. The NHHR has emerged as a valuable metric, offering a comprehensive assessment by accounting for both HDL and non-HDL levels. Therefore, this study focused on investigating the NHHR and its association with NAFLD in individuals with gout.

**Table 2:** The risk factors of NAFLD among gout patients.

Variables	B	SE	Wald *χ*^2^	OR (95% CI)	*p* value
Sex					
Female				1	
Male	0.147	0.425	0.120	1.159 (0.503–2.667)	0.729
Smoking					
No				1	
Yes	−0.237	0.127	3.478	0.789 (0.615–1.012)	0.062
Drinking					
No				1	
Yes	−0.286	0.133	4.645	0.752 (0.580–0.974)	0.031
Kidney stones					
No				1	
Yes	−0.11	0.174	0.404	0.895 (0.637–1.259)	0.525
Febuxostat					
No				1	
Yes	−0.298	0.127	5.488	0.742 (0.578–0.952)	0.019
Allopurinolo					
No				1	
Yes	−0.218	0.268	0.666	0.804 (0.476–1.358)	0.414
Benzbromarone					
No				1	
Yes	0.281	0.181	2.395	1.324 (0.928–1.890)	0.122
Age	−0.049	0.005	101.238	0.952 (0.943–0.961)	<0.001
Duration of gout	−0.07	0.01	49.515	0.932 (0.914–0.951)	<0.001
BMI	0.292	0.024	147.969	1.339 (1.277–1.403)	<0.001
SBP (mmHg)	0.002	0.004	0.403	1.002 (0.995–1.009)	0.525
DBP (mmHg)	0.017	0.005	9.287	1.017 (1.006–1.028)	0.002
CRP	−0.008	0.002	19.376	0.992 (0.988–0.995)	<0.001
FPG (mmol/L)	0.015	0.071	0.043	1.015 (0.883–1.166)	0.836
TC (mmol/L)	0.356	0.066	29.013	1.428 (1.255–1.626)	<0.001
LDL-C (mmol/L)	0.415	0.086	23.473	1.515 (1.281–1.792)	<0.001
HDL-C (mmol/L)	−0.341	0.233	2.136	0.711 (0.450–1.123)	0.144
TG (mmol/L)	0.605	0.077	61.938	1.831 (1.575–2.128)	<0.001
eGFR	0.018	0.002	60.525	1.019 (1.014–1.023)	<0.001
UA (umol/L)	0.002	0.001	9.911	1.002 (1.001–1.003)	0.002
ALT (U/L)	0.02	0.003	60.289	1.020 (1.015–1.025)	<0.001
AST (U/L)	0.029	0.006	27.965	1.030 (1.019–1.041)	<0.001
GGT (U/L)	0.003	0.001	5.432	1.003 (1.001–1.006)	0.020
ALP (U/L)	−0.012	0.003	23.136	0.988 (0.983–0.993)	<0.001
TSH	0.025	0.029	0.733	1.025 (0.969–1.084)	0.392
FT4	0.018	0.024	0.562	1.018 (0.971–1.068)	0.453
FT3	0.522	0.082	40.928	1.686 (1.436–1.978)	<0.001
Phosphorus (mmol/L)	1.496	0.335	19.929	4.462 (2.314–8.604)	<0.001
Magnesium (mmol/L)	1.305	0.621	4.414	3.687 (1.091–12.457)	0.036
Sodium (mmol/L)	−0.006	0.025	0.049	0.995 (0.947–1.044)	0.825
Potassium (mmol/L)	−0.781	0.162	23.104	0.458 (0.333–0.630)	<0.001
NHHR	0.297	0.062	23.180	1.346 (1.193–1.519)	<0.001

BMI, body mass index; SBP, systolic blood pressure; DBP, diastolic blood pressure; HbA1c, glycated hemoglobin; FPG, fasting plasma glucose; TC, total cholesterol; LDL-C, low-density lipoprotein cholesterol; HDL-C, high-density lipoprotein cholesterol; TG, triglycerides; UACR, urine microalbumin/creatinine; eGFR, estimated glomerular filtration rate; UA, uric acid; ALT, alanine aminotransferase; AST, aspartate aminotransferase; GGT, γ-glutamyltransferase; ALP, alkaline phosphatase; TSH, thyroid-stimulating hormone; FT4, free thyroid hormone; and FT3, free triiodothyronine. positive associations (OR > 1), negative association (OR < 1).

### 3.3 The relationship between NHHR and NAFLD among individuals suffering from gout

To study the connection between NHHR and NAFLD in gout, multivariate logistic regression was utilized (Table 3). According to the unadjusted Model 1, NHHR significantly increased the risk of NAFLD in individuals with gout, with an OR of 1.346 [95% confidence interval (CI): 1.193–1.519, *p* < 0.001]. In the partially adjusted Model 2, a positive correlation between NHHR and NAFLD in individuals with gout was observed, with an OR of 1.251 (95% CI: 1.101–1.418, *p* = 0.001). After accounting for all variables in Model 3, the positive correlation persisted as statistically significant, with an OR of 1.242 (95% CI: 1.089–1.416, *p* = 0.001). The results indicated that for each unit increase in NHHR, there was a 24.2% rise in the probability of NAFLD prevalence in people with gout.

**Table 3:** The association between NHHR and NAFLD in gout patients.

Exposure	Model 1		Model 2		Model 3	
	OR (95% CI)	*p* value	OR (95% CI)	*p* value	OR (95% CI)	*p* value
NHHR	1.346 (1.193–1.519)	<0.001	1.25 L (1.101–1.418)	0.001	1.242 (1.089–1.416)	0.001
NHHR group [median (range)]						
Q1 2.25 [≤2.69]	Ref.		Ref.		Ref.	
Q2 2.98 [2.70–3.33]	1.830 (1.290–2.597)	0.001	1.839 (1.270–2.663)	0.001	1.823 (1.249–2.661)	0.002
Q3 3.66 [3.34–4.07]	2.105 (1.481–2.992)	<0.001	1.996 (1.378–2.892)	<0.001	1.938 (1.329–2.826)	0.001
Q4 4.65 [≥4.08]	2.456 (1.718–3.512)	<0.001	2.022 (1.387–2.949)	<0.001	1.993 (1.349–2.944)	0.001
*P* trend	<0.001		<0.001		<0.001	

OR odds ratio, CI confidence interval. Model 1 were not adjusted. Model 2 were adjusted for age. Model 3 was further adjusted for UA, phosphorus, magnesium and eGFR.

By converting the NHHR variable into quartiles from its continuous form, the three models that used NHHR as a categorical variable revealed a significant increase in NAFLD prevalence in gout with increased NHHR (*p* for trend < 0.001). In Model 1, the OR with 95% CI for the prevalence of NAFLD in the second, third, and fourth quartiles of the NHHR, relative to the first quartile, were 1.830 (95% CI:1.290–2.597, *p* = 0.001), 2.105 (95% CI:1.481–2.992, *p* < 0.001), and 2.456 (95% CI:1.718–3.512, *p* < 0.001), respectively. In Model 2, the corresponding ORs (95% CI) were 1.839 (95% CI: 1.270–2.663, *p* = 0.001), 1.996 (95% CI: 1.378–2.892, *p* < 0.001), and 2.022 (95% CI: 1.387–2.949, *p* < 0.001), respectively. In Model 3, the ORs (95% CI) for NAFLD prevalence in the second, third, and fourth NHHR quartiles, as compared to the first quartile, were 1.823 (95% CI: 1.249–2.661, *p* = 0.002), 1.938 (95% CI: 1.329–2.826, *p* = 0.001), and 1.993 (95% CI: 1.349–2.944, *p* = 0.001), respectively.

### 3.4 Nonlinear connections between NHHR and NAFLD in individuals with gout

**Figure 2:**
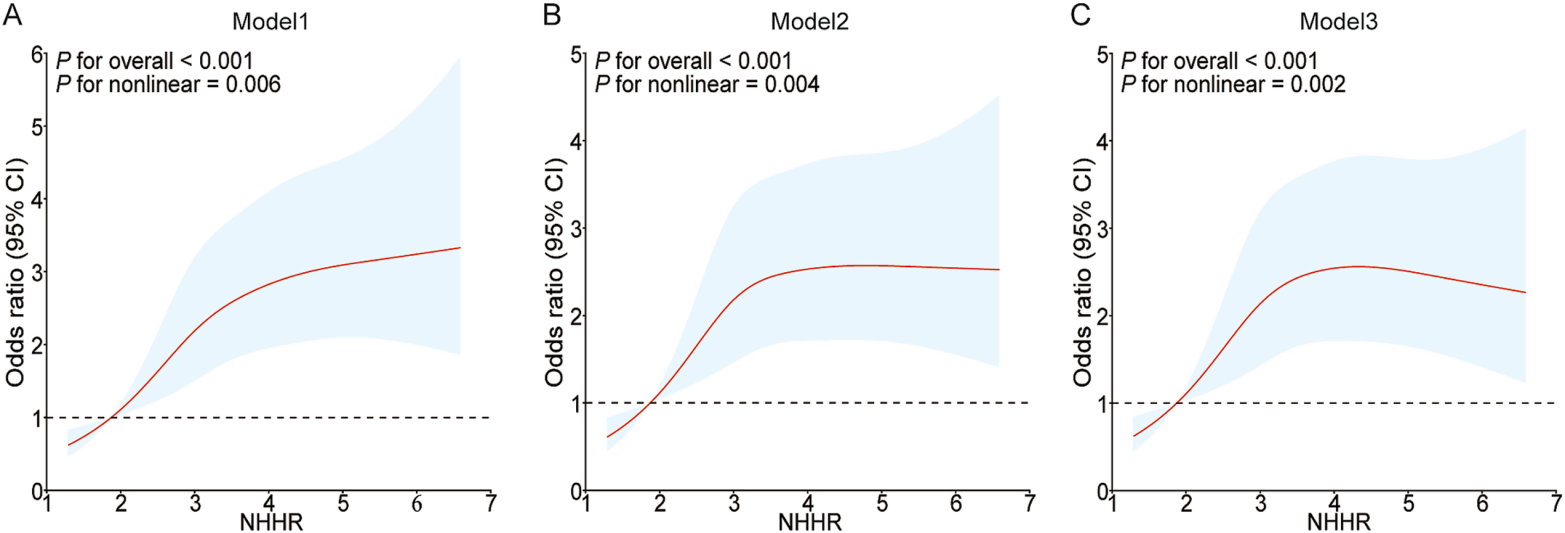
Restricted cubic spline curves for NAFLD risk in gout according to the NHHR. The association between NHHR and NAFLD risk in gout patients is depicted by the RCS curves in Model 1 **(A)**, Model 2 **(B)**, and Model 3 **(C)**.

To examine the possible nonlinear association between NHHR and NAFLD in people with gout, the RCS curve model was applied. A nonlinear dose–response relationship was noted between NHHR and NAFLD in all models, whether they were unadjusted, partially adjusted, or fully adjusted, due to nonlinearity *p*-values being under 0.05. In addition, with the help of the ‘segmented’ package, we discovered that the inflection points for NHHR regarding NAFLD risks in gout were 2.054, 2.104, and 1.927 in the respective models (Figure 2). We also assessed the effect sizes and confidence intervals on both sides of the inflection point 1.927. The effect size was 2.206 (95% CI: 0.241–20.191, *p* = 0.484) to the left of the inflection point, and 1.182 (95% CI: 1.022–1.368, *p* = 0.025) to the right (Figure 2C; Table 4).

**Table 4:** The result of the two-piecewise linear regression model.

Outcome	Effect	*p* value
Model 1 Fitting model by standard linear regression	1.242 (1.089–1.416)	0.001
Model 2 Fitting model by two-piecewise linear regression		
Inflection point	1.927	
<1.927	2.206 (0.241–20.191)	0.484
≥1.927	1.182 (1.022–1.368)	0.025
*p* for likelihood test		<0.001

### 3.5 Subgroup analysis

**Figure 3:**
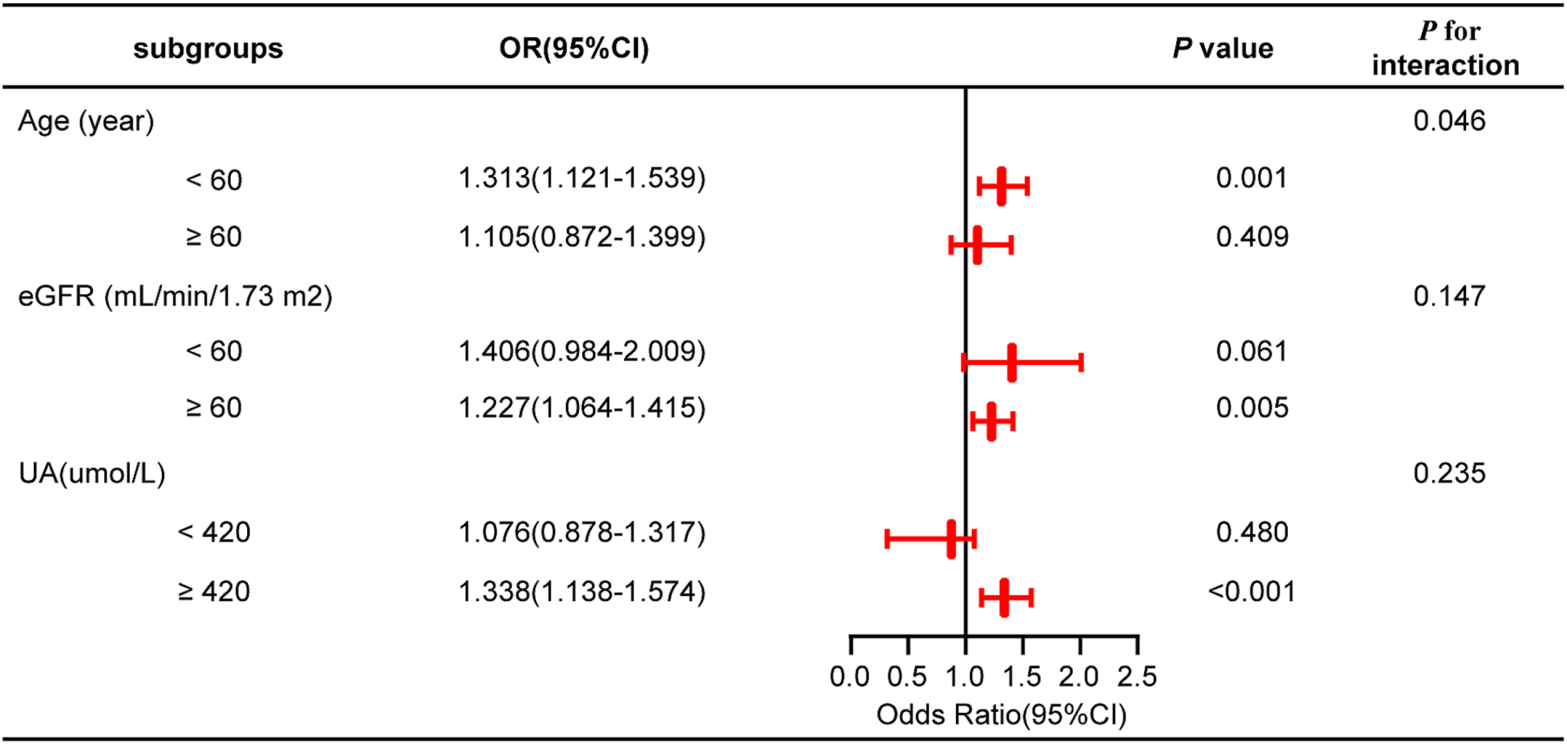
Subgroup analysis for the association of NHHR and NAFLD risk in gout.

A subgroup analysis was conducted treating NHHR as a continuous variable to further investigate its impact on outcome measures across subgroups stratified by age, eGFR, and UA levels. The findings indicated that none of these covariates significantly modified the connection between NHHR and NAFLD in gout patients, as evidenced by interaction *p*-values exceeding 0.05 (Figure 3; Supplementary Table 1).

### 3.6 The potential of NHHR to forecast NAFLD risk in gout patients

**Figure 4:**
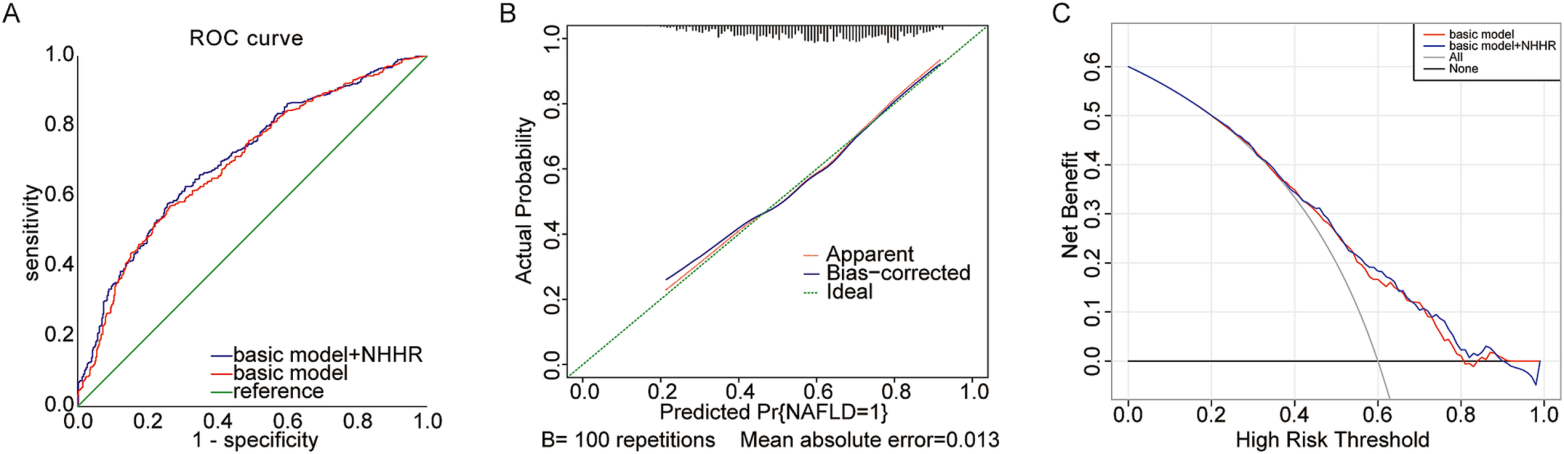
**(A)** Predictive utility test of NHHR for NAFLD risk in gout The area under the receiver operating characteristic (ROC) curve (AUC); **(B)** Calibration curve; **(C)** Decision curve analysis (DCA).

The foundational model incorporated variables including age, UA, phosphorus, magnesium, and eGFR. The novel variable NHHR was subsequently added to this basic model, and the diagnostic capabilities of both models were analyzed using ROC curves. Initially, the area under the curve (AUC) was 0.696 (95% CI 0.663–0.729), but it rose to 0.706 (95% CI 0.674–0.739) after NHHR was included (Figure 4A; Table 5). The findings from the calibration curve analysis indicated that there was only a minimal deviation between the ideal and actual curves. The empirical predictive curve closely aligned with the ideal curve (B = 100 repetitions, Mean Absolute Error = 0.013), suggesting that the prediction model exhibited a high degree of accuracy (Figure 4B). Decision Curve Analysis (DCA) was employed to assess the model’s clinical utility, demonstrating a favorable net benefit and a positive impact on clinical practice (Figure 4C).

**Table 5:** The AUC of basic model and NHHR incremental model in predicting NAFLD risk in gout patients.

Variable	Cut-off	AUC	95% CI	*p* value	J-Youden	Sencitivity%	Specificity%	PPV	NPV
Basic model	0.627	0.696	0.663–0.729	<0.001	0.308	0.572	0.736	0.54	0.76
+NHHR	0.629	0.706	0.674–0.739	<0.001	0.322	0.581	0.741	0.54	0.77

## 4 Discussion

This research employed a cross-sectional approach to explore the link between NHHR and the likelihood of NAFLD in people with gout. We observed a positive link between NHHR and the occurrence of NAFLD among gout patients, even after accounting for different covariates. A non-linear connection between NHHR and NAFLD was also observed in those suffering from gout. Furthermore, the correlations between NHHR and NAFLD in different subgroups were in line with the overall study population results. Finally, our study found that a higher NHHR was associated with a greater risk of NAFLD in individuals with gout.

The prevalence of gout and NAFLD has risen in recent years, attributed to the global spread of type 2 diabetes, obesity, and increased uric acid levels. Four commonalities indicate a significant relationship between gout and NAFLD: both are more frequently observed in men than women, both are prevalent among those with chronic illnesses, both are closely tied to metabolic disorders, and both are associated with dietary patterns (1, 19, 20). It is challenging to identify the causal relationship between gout and NAFLD due to their overlapping pathogenesis and risk factors. However, many studies consistently indicate that gout elevates the chances of developing NAFLD, and the increase in NAFLD is connected to gout (21–23). Therefore, it was highly important to identify risk factors for NAFLD development in gout patients.

This is the first study to investigate the association between NHHR and NAFLD specifically in gout patients. Dyslipidemia is a widely recognized risk factor for NAFLD (24). The research utilized a nested case–control design, including 98 patients with NAFLD and 100 healthy individuals, all part of a longitudinal study conducted in Mexico. The study revealed significant differences between the NAFLD cases and the control group in terms of total serum levels of lysophosphatidylcholines, sphingomyelins, triacylglycerols, and cholesterol esters (25). In another cohort study from China with 12,126 participants, researchers employed a joint index of HDL-C and GGT to explore their connection to NAFLD. Using the GGT/HDL-C ratio was more effective for predicting NAFLD than using HDL-C alone (26). Furthermore, research involving 14,251 individuals also indicated that the triglyceride glucose-body mass index is a better predictor of NAFLD compared to the conventional lipid parameters TC, TG, and HDL-C (27). In conclusion, A growing body of research endorses the use of novel complex lipid-related markers for predicting NAFLD.

The novel lipid ratio marker, NHHR, is both cost-effective and widely accessible. Prior investigations have demonstrated a significant relationship between NHHR and the risk of dying from any cause or cardiovascular disease among U. S. adults who have diabetes or prediabetes (28). Likewise, another study has uncovered a robust association between NHHR and an elevated risk of hyperuricemia (HUA) (29). Moreover, analysis of data from the NHANES survey, spanning 2001 to 2018, showed a steady positive relationship between NHHR and hypertension (30). Consequently, the substantial link between NHHR and these risks of metabolic diseases provides indirect support for our research findings.

Herein, we identified a notable positive correlation between NHHR and the incidence of NAFLD among individuals with gout (OR:1.242 95% CI: 1.089–1.416). This result is supported by some research. A cross-sectional analysis of data from 3,529 participants in the National Health and Nutrition Examination Survey (NHANES) for the years 2017–2020 identified a notable positive link between NHHR and NAFLD, even after accounting for confounding variables (OR: 1.33, 95% CI: 1.24–1.42). This study also demonstrates a non-linear connection between NHHR and NAFLD (31). Similarly, the analysis encompassed 3,784 participants from the 2017–2018 NHANES, revealing a consistently positive association between NHHR and Liver disease linked to metabolic dysfunction in populations with obesity or type 2 diabetes (OR = 1.26, 95% CI = 1.18–1.35) (32). Moreover, a prospective cohort study involving 12,648 adult NAFLD patients from the NHANES database (1999–2018) revealed that NHHR is linked to all-cause mortality in NAFLD patients, showing unique non-linear associations (33). These studies aim to explore the connection between NHHR and NAFLD among the general populace, as well as among individuals with obesity and type 2 diabetes. This investigation is the first to systematically analyze and present this association in a population of patients with a definitive gout diagnosis. Gout patients, with their considerable hyperuricemia, chronic inflammatory condition, and specific lipid metabolism characteristics, are at a greater risk of NAFLD. Our research addresses a previously unexamined area within the existing literature concerning this significant and distinct clinical population. It offers novel empirical evidence to enhance the understanding of lipid metabolism-related factors that contribute to the risk of NAFLD in patients with gout. Furthermore, our findings propose that NHHR could be a valuable marker for risk stratification of NAFLD in this population. Future investigations are encouraged to delve deeper into the underlying mechanisms of this association in gout patients and to assess its implications for clinical management.

In addition, the RCS results indicate a non-linear connection between NHHR and NAFLD. According to the results, an NHHR value of 1.927 might be a possible intervention threshold for NAFLD in individuals suffering from gout. Given this threshold, we can suggest specific nutritional and exercise interventions to reduce the risk of NAFLD in those with gout. Food-derived lipids are generally an important source of lipids for the human body (34). Consequently, an appropriate dietary plan is important for the prevention and management of NAFLD in gout patients. Prior investigations have demonstrated that consuming a low-fat diet can markedly improve NAFLD and gout (35, 36). Despite the difficulty in forming a universal dietary standard for NAFLD in gout patients, doctors can still offer advice based on the principle of a low-fat diet in clinical practice. Additionally, exercise therapy might be an essential strategy for managing gout-related NAFLD. Prior investigations have revealed that physical exercise can markedly improve irregular blood lipid profiles (37). Studies conducted previously have also demonstrated that enhancing physical activity may decrease the risk of gout and NAFLD (38, 39). Because of the unique and varied differences in time, intensity, and exercise type among NAFLD patients with gout, creating a universally applicable standard for exercise therapy is challenging. Further investigation into various exercise methods tailored for NAFLD patients with gout is essential, considering factors like gender, age, disease activity, and lifestyle.

We have put forward several theoretical explanations concerning the impact of NHHR on the onset and progression of NAFLD, based on current studies. NAFLD is characterized by enhanced liver uptake and new fat creation, yet the compensatory boost in fatty acid oxidation fails to normalize lipid levels. This deficiency might worsen cellular damage and accelerate disease progression by causing oxidative stress, especially when mitochondrial function is impaired and there is increased oxidation in peroxisomes and cytochrome systems (40). Lipid metabolism and oxidative stress are crucial in the development of gout. Lipid modifications and oxidative stress in individuals with gout are typically noticeable in clinical settings and have a strong connection to uric acid metabolism and inflammation (41–43). Patients with gout have increased levels of TG and LDL-C (44, 45). An increase in NHHR suggests higher levels of non-HDL-C, especially LDL-C, which can undergo oxidation to form oxidized low-density lipoprotein (ox-LDL) (46). Ox-LDL leads to oxidative stress and mitochondrial issues in liver cells, aiding in the progression of NAFLD (47, 48). Moreover, gout patients experience a decrease in HDL-C levels (45), which weakens antioxidant capabilities and increases the likelihood of LDL oxidation (49). One of the biggest risk factors for NAFLD is insulin resistance, which is closely linked to NHHR and contributes to liver steatosis (50, 51). These evidences further validate that NHHR is strongly linked to the occurrence of NAFLD and serves as a dependable indicator of NAFLD risk in individuals with gout.

This study was conducted in a real-world environment with a significant number of participants and is the first to investigate the connection between NHHR and NAFLD in individuals with gout. The research utilized comprehensive multivariable regression analyses, considering various confounding variables such as age, UA, phosphorus, magnesium, and eGFR, thereby reducing potential bias in the findings. However, the study has certain limitations. The study concentrated on patients who were hospitalized, which might restrict how the findings apply to other groups. Future research should focus on enhancing generalizability by clearly recruiting participants from a range of settings, including primary care clinics and community cohorts. Conducting multi-center research involving hospitals treating gout could be crucial in verifying and expanding our observations to a broader group of gout sufferers. Furthermore, the study’s cross-sectional design cannot infer a definitive causal relationship between NHHR and NAFLD in individuals diagnosed with gout. Addressing this requires longitudinal cohort studies. By following gout patients over time to examine NAFLD development in relation to baseline and NHHR variations, stronger evidence for potential causal links could be established. Additionally, trials that focus on changing NHHR, for instance, via lifestyle adjustments or lipid-lowering therapies, and then assessing the outcomes on NAFLD, would be crucial for proving causality and exploring treatment options. Although numerous confounding factors affecting NHHR and NAFLD in this population were considered, some potential confounders, such as dietary habits, daily routines, and genetic traits, were not included. Additionally, the dataset does not include data on lipid-lowering drugs, which might influence LDL-C levels and NHHR, possibly causing residual confounding. Future investigations ought to emphasize the integration of detailed data on these absent factors. This process entails employing datasets that have complete electronic health records and collecting detailed lifestyle and dietary information ahead of time, with meticulous documentation of medication.

## 5 Conclusion

We propose that a higher NHHR is linked to an increased risk of NAFLD. These results indicate that NHHR could be used clinically to identify people with gout who are at an increased risk for NAFLD, which may help in early screening and risk assessment strategies. Nonetheless, it is crucial to validate this prospectively in various cohorts to ensure its usefulness before it is widely implemented in clinical settings.

## Data Availability

The raw data supporting the conclusions of this article will be made available by the authors, without undue reservation.
