# Supplementary material for: Assessing the association between the non-HDL to HDL cholesterol ratio and NAFLD in Chinese adults: concentrate on gout populations
Source: Front Nutr. 2025 Sep 1;12:1655817. doi: 10.3389/fnut.2025.1655817 (PMC12433996; doi:10.3389/fnut.2025.1655817)
Supplement: Supplementary file 1 [file Table_1.DOCX]

**Supplementary Table 1 Exploring subgroup associations between NHHR and the risk of NAFLD in people with gout**

| **subgroups** | **OR(95%CI)** | ***P* value** | ***P* for interaction** |
| --- | --- | --- | --- |
| Age (year) |  |  | 0.046 |
| ＜60 | 1.313(1.121-1.539) | 0.001 |  |
| ≥60 | 1.105(0.872-1.399) | 0.409 |  |
| eGFR (mL/min/1.73 m2 ) |  |  | 0.147 |
| ＜60 | 1.406(0.984-2.009) | 0.061 |  |
| ≥60 | 1.227(1.064-1.415) | 0.005 |  |
| UA(umol/L) |  |  | 0.235 |
| ＜420 | 1.076(0.878-1.317) | 0.480 |  |
| ≥420 | 1.338(1.138-1.574) | ＜0.001 |  |
